# Genetics of Degenerative Cervical Myelopathy: A Systematic Review and Meta-Analysis of Candidate Gene Studies

**DOI:** 10.3390/jcm9010282

**Published:** 2020-01-20

**Authors:** Daniel H. Pope, Benjamin M. Davies, Oliver D. Mowforth, A. Ramsay Bowden, Mark R. N. Kotter

**Affiliations:** 1 School of Clinical Medicine, University of Cambridge, Cambridge CB2 0SP, UK; 2 Division of Neurosurgery, Department of Clinical Neurosciences, University of Cambridge, Cambridge CB2 0QQ, UK; 3 Department of Clinical Genetics, Cambridge University Hospitals NHS Foundation Trust, Cambridge CB2 0QQ, UK; 4 The Wellcome Trust/Cancer Research UK Gurdon Institute and Department of Biochemistry, University of Cambridge, Cambridge CB2 1QN, UK; 5 Anne McLaren Laboratory for Regenerative Medicine, Wellcome-MRC Cambridge Stem Cell Institute, University of Cambridge, Cambridge CB2 0SZ, UK

**Keywords:** genetics, single nucleotide polymorphism, degenerative cervical myelopathy, ossification posterior longitudinal ligament, severity, surgery

## Abstract

Degenerative cervical myelopathy (DCM) is estimated to be the most common cause of adult spinal cord impairment. Evidence that is suggestive of a genetic basis to DCM has been increasing over the last decade. A systematic search was conducted in MEDLINE, EMBASE, Cochrane, and HuGENet databases from their origin up to 14th December 2019 to evaluate the role of single genes in DCM in its onset, clinical phenotype, and response to surgical intervention. The initial search yielded 914 articles, with 39 articles being identified as eligible after screening. We distinguish between those contributing to spinal column deterioration and those contributing to spinal cord deterioration in assessing the evidence of genetic contributions to DCM. Evidence regarding a total of 28 candidate genes was identified. Of these, 22 were found to have an effect on the radiological onset of spinal column disease, while 12 genes had an effect on clinical onset of spinal cord disease. Polymorphisms of eight genes were found to have an effect on the radiological severity of DCM, while three genes had an effect on clinical severity. Polymorphisms of six genes were found to have an effect on clinical response to surgery in spinal cord disease. There are clear genetic effects on the development of spinal pathology, the central nervous system (CNS) response to bony pathology, the severity of both bony and cord pathology, and the subsequent response to surgical intervention. Work to disentangle the mechanisms by which the genes that are reviewed here exert their effects, as well as improved quality of evidence across diverse populations is required for further investigating the genetic contribution to DCM.

## 1. Introduction

Degenerative cervical myelopathy (DCM) is estimated to be the most common cause of spinal cord impairment in the adult population and its incidence is expected to rise as the population continues to age [1]. The term DCM is relatively new, and it was proposed to unify degenerative pathologies with a common injury mechanism (subacute, progressive spinal cord injury) and treatment (decompressive surgery) [1]. This includes both cervical spondylosis (such as degenerative disc disease or osteophyte formation) and the ossification of the posterior longitudinal ligament (OPLL) or ligamentum flavum (OLF) [1,2,3,4]. These aetiologies were often previously separately considered, as cervical spondylotic myelopathy (CSM) and OPLL.

The trajectory of DCM between patients is heterogenous and currently unpredictable and unexplained [3]. For example, mechanical compression is an imaging hallmark of the disease. However, the location and amount of compression does not correlate with the disease symptoms [5,6,7]. In fact, the clinical phenotype can range from asymptomatic to severe disability, nearly independent from the amount of compression. Furthermore, patients’ response to surgical decompression, the mainstay of treatment, is variable: it achieves excellent improvements in some patients, whereas in others these do not occur [8]. Such variation between patients has led to increasing interest in the genetic basis of this condition. One study reported a relative risk of 5.21 for the development of DCM in first-degree relatives of patients [9].

So far, the effects of genes involved in inflammation, bone, and lipid metabolism have been linked to both the pathogenesis of DCM and the response to surgical intervention [10,11]. However, these studies have failed to disentangle their relationship to spinal degeneration and myelopathy. This is important, as the fact that symptom progression and severity of spinal cord compression correlate poorly suggests that the genetic polymorphisms that contribute to spinal column degeneration may be distinct from those that influence the development of myelopathy in response to the resulting spinal cord compression.

Moreover, reviews have focused on CSM or OPLL, as opposed to DCM. Genes that influence how the spinal cord copes with mechanical stress may be identifiable in studies that investigate the severity of myelopathy and, in particular, the response to surgery.

Therefore, the objectives of this review are to provide a synthesis of the published literature on a genetic contribution to the susceptibility to develop degenerative spinal column changes that lead to DCM, the heterogeneity in severity of the clinical manifestation of DCM, and the heterogeneity in response to surgery, in order to evaluate the genes that are specifically linked to the onset and recovery of myelopathy.

## 2. Methods

A systematic review was conducted in accordance with the PRISMA guidelines; a PRISMA checklist is presented in the Supplementary Data [12]. A search was conducted in MEDLINE, EMBASE, Cochrane, and HuGENet databases for all relevant papers from database origin up to 14th December 2019. The full search strategy is presented in the Supplementary Data and it was developed in conjunction with the Medical Library at the University of Cambridge School of Clinical Medicine. Reference lists of key articles were systematically examined to identify further eligible articles.

Titles and abstracts were screened for relevance and, subsequently, full text papers were screened for eligibility, according to the following inclusion criteria:

- Primary clinical trial
- DCM is the primary condition being addressed
- Focus on genetics (specific gene identified)
- Human study
- English language
- Full text article

Animal studies, case reports, letters, editorials, reviews, technical notes, commentaries, proposals, and corrections were excluded. In addition, articles meeting the following criteria were excluded:

- Paediatric studies (patients < 18 years)
- Focus on acute trauma and acute spinal cord injury
- Focus on thoracic or lumbar spine

Two authors independently assessed the full-texts of potentially relevant articles (DHP and BMD), with any disagreements being resolved through discussion until agreement was reached.

Data that were extracted from the eligible articles included: study design, number of cases, number of controls, participant demographics, patient disease profile, gene studied, polymorphism/haplotype studied, and effects of polymorphisms and haplotypes on DCM susceptibility/severity/response to surgery (principal summary measures: odds ratios). The risk of bias was assessed through an evaluation of study design, methods of study population selection, matching of controls to cases, and the consideration of publication source. The MINORS methodological items were used to give structure to this process [13]. The GRADE guidelines were used to rate the quality of evidence for each candidate gene, and across genes for each of the three main questions (susceptibility, severity, response) [14].

Meta-analysis using the Cochrane Review Manager 5.3 software was used for polymorphisms, where more than one study had investigated the same polymorphism and the requisite data were available.

## 3. Results

**Figure 1:**
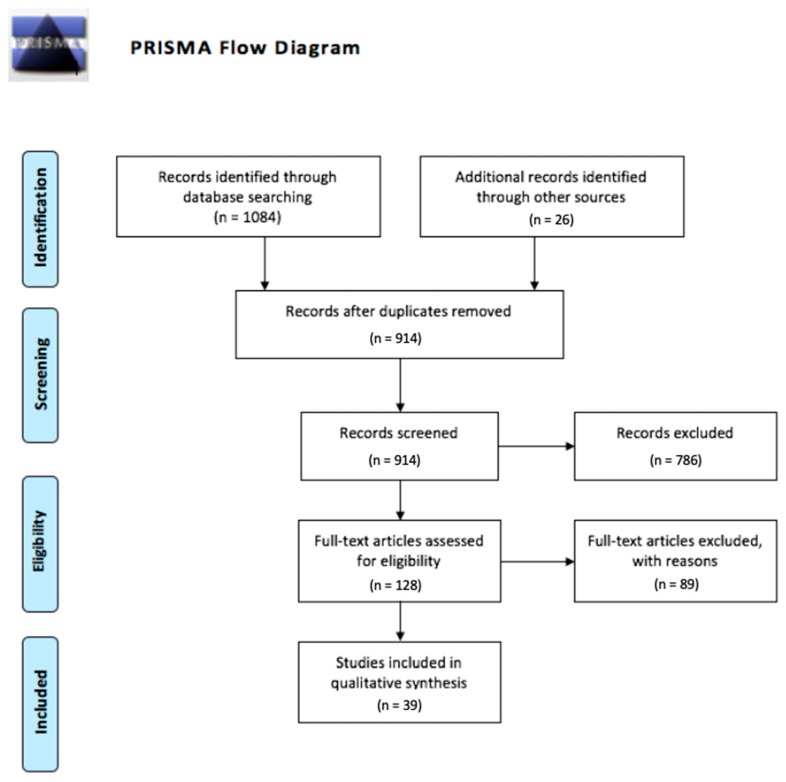
PRISMA flow diagram of search and screening.

After removing duplicates, a total of 914 articles were screened and 39 were eligible for inclusion (Figure 1). In total, 37 articles addressed the genetics of susceptibility to developing DCM, 13 articles addressed the genetics of heterogeneity in DCM severity (either radiological or clinical severity) and six addressed the genetics of response to surgery. A total of 28 genes were identified, with key information regarding each candidate gene presented in Tables 1–3.

### 3.1. What are the Genetic Effects on Susceptibility to Development of DCM?

Evidence regarding the onset of DCM/OPLL was identified for 28 genes: ACE, APOE, BID, BMP2, BMP4, BMP9, COL6A1, COL9A2, COL11A2, FGF2, FGFR1, FGFR2, HIF1A, IL1B, IL15RA, IL18RAP, leptin receptor, NPPS, OPG, OPN, RUNX2, TGFB1, TGFB3, TGFBR2, TLR5, VDBP, VDR, and VKORC1. Of these 28 genes, 22 were found to be associated with the radiological onset of spinal pathology, while 12 were associated with the clinical development of DCM (i.e., spinal cord pathology). For six genes, no significant effect of polymorphisms has been found by the studies reviewed to date: FGF2, FGFR2, IL18RAP, leptin receptor, TLR5, and VDBP. Most of the genes (19, 68%) have been investigated by only a single study. Bone morphogenetic protein genes (9, 32%) and collagen genes were the most studied gene groups (8, 29%). Table 1 presents full information for each gene.

#### 3.1.1. Spinal Pathology

The majority of studies investigating the genetics of susceptibility to DCM used the radiological definition of cases. Therefore, these studies assess the development of bony spinal pathology (an initial stage in overall DCM development).

Kim et al. (2014) investigated the *ACE* gene, finding the deletion/deletion genotype of the intron 16 polymorphism (rs4646994) to be associated with an increased risk of developing radiological OPLL (AOR 2.20, *p* = 0.002) [15]. Similarly, two SNPs of the *BID* gene (rs8190315, rs2072392) were associated with the development of OPLL (OR 2.66, *p* = 0.005 for both) [18].

Four studies have investigated the role of variants in *BMP2*. Wang et al. (2008) found no significant effect of the Ser87Ser SNP, but found the Ser37Ala SNP was associated with an increased risk of OPLL development (*p* < 0.001) [19]. Interestingly, however, patients with the GG genotype of Ser87Ser had significantly greater number of ossified vertebrae, which suggested the A allele restricts ectopic ossification in OPLL. Meanwhile, the Ser37Ala SNP had no significant effect on the number of ossified vertebrae.

Yan et al. (2013) also found the Ser37Ala SNP to be associated with increased risk (*p* < 0.001) [21], although a more recent study that compared OPLL patients to their family members found no effect of either the Ser87Ser or Ser37Ala SNPs on risk of OPLL (*p* = 0.411, *p* = 0.670, respectively) [22]. Additionally, the 570A>T SNP in the *BMP2* gene was not found to be significantly associated with risk of OPLL [21]. Liu et al. (2010) used a patient cohort that included OPLL, OLF, and OPLL + OLF patients, but found no effect of the rs1005464 intronic SNP on the susceptibility of radiological DCM development [20].

In the *BMP4* gene, the 6007C>T SNP was found to be associated with an increased risk of developing radiological OPLL in male patients (OR 1.57, *p* = 0.014), although the effect is lost when males and females are considered together (*p* = 0.493) [23]. In the same SNP, the CT and TT genotypes were associated with a greater number of ossified vertebrae (*p* = 0.043) [23], as was a haplotype (TGGGCTT) containing seven SNPs (*p* = 0.002). Ren et al. (2012a) identified three SNPs that significantly increase the risk of OPLL: rs54419150 (OR 3.48, *p* < 0.001), rs17563 (OR 2.22, *p* < 0.001), and rs76335800 (OR 1.99, *p* < 0.001). Linkage disequilibrium studies also identified the haplotype block TGGGCTT containing these three SNPs to be significantly associated with the occurrence of OPLL (OR 2.54, *p* < 0.001) [24].

In the *BMP9* gene, two SNPs and a haplotype containing four SNPs were found to be associated with an increased risk of OPLL development: rs75024165 (OR 1.82, *p* < 0.001), rs34379100 (OR 1.95, *p* = 0.003), and haplotype CTCA (OR 2.37, *p* < 0.001). The haplotype was also associated with development of a greater number of ossified vertebrae (*p* = 0.001). A further SNP (rs9421799) was found to be protective (OR 0.69, *p* = 0.004), while three SNPs had no significant effect [26].

Wang et al. (2018) investigated the *BMPR1A* gene, finding two SNPs (-349C>T, 4A>C) that were associated with an increased risk of OPLL development (*p* < 0.001 both), and two (1327C>T, 1395G>C) with no significant effect [27]. Furthermore, patients with the C allele of the 4A>C SNP were more likely to have a greater number of ossified vertebrae on lateral cervical radiograph (*p* < 0.001).

The *COL6A1* gene has been the subject of four studies. Tanaka et al. (2003) investigated 32 SNPs in the *COL6A1* gene, of which 21 were significantly associated with OPLL (see Table 1) [28]. Further work by Kong et al. (2007) was consistent with these findings, with intron 32 (-29) C allele conferring a greater risk of OPLL (OR 1.89, *p* = 0.004) [29]. However, Liu et al. (2010) reported no significant effect of the rs2276255 SNP on the risk of OPLL or OLF development [20], in contrast to Tanaka et al.’s finding of a weak significant effect (*p* = 0.048). Further contradiction in the *COL6A1* gene is seen in Kong et al.’s (2007) finding that the promoter (−572) SNP T allele was associated with a 2.94 times greater risk of OPLL (*p* = 0.0003), while Kim et al. (2014) found no significant effect (*p* = 0.282) [22]. Liu et al. (2010) found no effect of one additional SNP (rs9978314) on the risk of OPLL or OLF development [20].

In the *COL11A2* gene, the intron 6 (−4) polymorphism was associated with a greater risk of OPLL development in two studies (OR 1.99, *p* = 0.0003; *p* = 0.0004) [31,32]. Similarly, the exon 6 (+28) polymorphism was associated with an odds ratio of 1.84 of developing OPLL (*p* = 0.0012) [32].

Jun & Kim (2012) investigated the *FGF2*, *FGFR1*, and *FGFR2* genes in 157 OPLL patients and 222 age- and sex-matched controls [34]. Three SNPs of the *FGF2* gene showed no significant effect on the likelihood of OPLL development, as did three SNPs of the *FGFR2* gene. However, the rs13317 SNP in the *FGFR1* gene was associated with an increased risk (OR 2.0, *p* = 0.02).

Kim et al. (2011) investigated two SNPs of the *IL15RA* (*IL15Rα*) gene [36]. The A allele of rs2228059 conferred a 1.52 times risk of radiological OPLL (*p* = 0.009), while the rs2296139 SNP had no significant effect.

The A861G polymorphism of the leptin receptor gene had no effect on the likelihood of OPLL development in a study of 156 OPLL patients and 93 age-matched controls [38].

In the *NPPS* gene, two studies both found no significant effect of the IVS20-11delT SNP on the likelihood of radiological OPLL (*p* = 0.512, *p* = 0.093) [38,41]. However, patients that were homozygous for the T deletion of the IVS20-11delT polymorphism had fewer ossified vertebrae and less thick ossification of their cervical vertebrae (*p* < 0.001 for both) [41].

The IVS15-14T>C and C973T SNPs were associated with an increased risk of radiological OPLL (*p* = 0.026, *p* < 0.001) [41]. Furthermore, patients with the T allele of the IVS15-14T>C SNP also had both a greater number of ossified vertebrae and greater thickness of ossification of their vertebrae (*p* < 0.001, *p* = 0.017, respectively). For the C973T SNP, the T allele was associated with increased thickness of ossified vertebrae (*p* = 0.007), but it had no effect on number of ossified vertebrae (*p* = 0.248). There was no effect of the A533C polymorphism on the likelihood of radiological OPLL development, or number of ossified vertebrae, or thickness of ossified vertebrae (*p* = 0.430, *p* = 0.363, *p* = 0.947) [41].

In a case-control study of OPLL, OLF, and OPLL+OLF patients, 11 SNPs of the *RUNX2* gene had no significant association with radiological development of OPLL/OLF [20]. However, patients with the C allele of the rs16873379 SNP had a greater number of ossified vertebrae (*p* = 0.001), as did patients with the A allele of the rs1406846 SNP (*p* = 0.020), and patients with the C allele of the rs2677108 SNP (*p* = 0.044).

**Figure 2:**
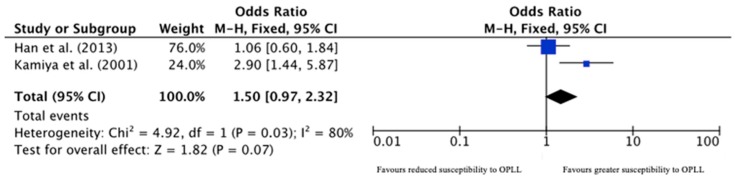
Forest plot for *TGFB1* 869T>C polymorphism.

In the *TGFB1 (TGFβ1)* gene, the CC genotype of the 869T>C polymorphism was found to be associated with an increased risk of radiological OPLL development in one study (OR 4.5, *p* = 0.0004) [45], but it had no such association in a recent study that involved almost double the number of cases (*p* = 0.656) [46]. On meta-analysis, there was no significant effect of the 869T>C polymorphism on the susceptibility to OPLL development (OR 1.50, 95% CI 0.97–2.32, *p* = 0.07; Figure 2). The 509C>T was found to have no association with radiological OPLL development [46].

Jekarl et al. (2013) investigated three SNPs of the *TGFBR2* (*TGFβR2*) gene, finding that two were associated with increased likelihood of OPLL development. The 445T>A polymorphism conferred a 2.81 times increased risk (*p* = 0.007), while the 571G>A polymorphism was associated with 8.73 times risk (*p* = 0.024) [47].

The *TLR5* gene has been investigated by one study, which found no association of three SNPs with the likelihood of OPLL development [48].

In the *VDR* gene, Kobashi et al. (2008) found the *FokI* polymorphism to be associated with 2.33 times increased risk of OPLL development (*p* = 0.0073) [50]. Similarly, Liu et al. (2010) found an association between the rs11574079 polymorphism and OPLL/OLF risk (OR 2.68, *p* = 0.0714) [20].

The *VKORC1* gene was investigated in 98 OPLL patients and 200 control subjects, with the −1639G> A polymorphism having a significant effect in female patients (OR 5.22, *p* = 0.004), but not when both sexes were considered together (*p* > 0.05) [52].

In the *NPPS* gene, He et al. (2013) examined the effect of four SNPs on the progression of OPLL ossification on lateral radiograph. The AA genotype of the A533C SNP and the homozygous T deletion genotype of the IVS20-11delT SNP were both associated with better responses to surgical intervention (OR 3.11, *p* = 0.029; OR 3.35, *p* = 0.007). The other two polymorphisms were not associated with any difference in response to surgery (good response defined as <2 mm increase in ossified mass of the posterior longitudinal ligament) [41].

#### 3.1.2. Spinal Cord Pathology

Multiple studies used clinical signs and symptoms of DCM alongside positive radiological findings. Such combination interrogates the development of cord pathology, rather than simply the development of spinal pathology.

In the *APOE* gene, the ε4 allele was found to be associated with an increased risk of myelopathy in a case-control study, where the controls had cervical spondylosis without myelopathy (OR 3.50, *p* = 0.008) [16]. However, a study in an Indian population found the ε2 allele to be associated with increased risk of myelopathy when compared to both the ε3 and ε4 alleles (OR 4.4, *p* = 0.002; OR 6.69, *p* = 0.009) [17].

In the *BMP4* gene, Wang et al. (2013) found the 6007C>T SNP to be protective for the development of clinical signs and symptoms of CSM (OR 0.51, *p* < 0.001) [25]. This is in contradiction to the evidence described above, in which this SNP was shown to be associated with an increased risk of radiological OPLL development [23,24].

The Trp2(+) allele of the *COL9A2* gene was associated with an increased risk of CSM development (OR 1.78, *p* = 0.048), a risk that was worsened by heavy smoking (OR 5.56, *p* < 0.001), while the Trp3 allele had no significant effect [30].

Koga et al. (1998) identified three polymorphisms of the *COL11A2* gene associated with DCM development: promoter (−182), exon 43 (+24) and exon 46 (+18) [31]. Horikoshi and colleagues investigated two additional SNPs of the *COL11A2* gene, but found no significant effect for either [33].

In the *HIF1A* (*HIF-1α*) gene, Wang et al. (2014) found no effect of the 1772C>T SNP, while the 1790G>A polymorphism was associated with an increased risk of CSM development (OR 1.62, *p* < 0.001) [35].

In the *IL15RA* gene, Guo et al. (2014) found a significant effect of the A allele of the rs2228059 SNP on DCM development (OR 1.63, *p* < 0.001) [37]. However, there was no effect of the rs2296139 SNP on the likelihood of developing symptomatic DCM. This is in commonality with the above findings of Kim et al. (2011) who showed rs2296139 had no effect on likelihood of developing radiological OPLL while the rs2228059 SNP did [36].

In the *IL18RAP* gene, Diptiranhan et al. (2019) found no significant effect of either the rs1420106 or rs917997 SNPs on the development of myelopathy (*p* > 0.05) [17].

Three studies have looked at the *NPPS* gene in relation to clinical onset of spinal cprd disease [33,39,40]. Nakamura et al. (1999) found the IVS20-11delT polymorphism to be associated with an increased risk of development of DCM (*p* = 0.0029) [39]. There is conflicting evidence of the effect of the IVS15-14T>C polymorphism: one study found it to be associated with a 3.01 times risk of myelopathy development (*p* = 0.022) [40], while another found no significant effect (*p* = 0.320) [33].

Yu et al. (2018) found no significant effect of the 1181G>C and 163A>G polymorphisms in the osteoprotegerin (*OPG*) gene, but found the C allele of the 950T>C SNP to be associated with a greater risk of myelopathy (*p* < 0.01) [42].

Wu et al. (2014) studied three SNPs of the osteopontin (*OPN*) gene [43]. Two showed no significant effect, but the G allele of the -66T>G SNP was associated with an odds ratio of 1.55 of clinical onset of DCM (*p* = 0.002).

In the *RUNX2* gene, Chang et al. (2017) found the SNPs rs967588 and rs16873379 to be protective for DCM development (OR 0.47, *p* = 0.033; OR 0.48, *p* = 0.033) [44]. The rs1406846 SNP was, on the other hand, strongly associated with DCM development (OR 5.67, *p* < 0.001). Four further SNPs had no significant effect.

Horikoshi et al. (2006) studied the *TGFB1* (*TGFβ1*) and *TGFB3* (*TGFβ3)* genes [33]. There was no significant effect of the IVS2+114G>A SNP of *TGFB1*, while the CC genotype IVS1-1284G>C SNP of *TGFB3* was associated with an increased risk of DCM development (OR 1.46, *p* = 0.044).

Song et al. (2018) found no significant effect of the Thr20Lys polymorphism of the *VDBP* gene (OR 0.973, *p* = 0.834) [49].

In the *VDR* gene, Wang et al. (2010) found no significant effect of *FokI* polymorphism on CSM risk [51]. The *BsmI* polymorphism also had no effect on CSM risk, but the *ApaI* and *TaqI* polymorphisms conferred a 2.88 times and 4.67 times increased CSM risk (both *p* < 0.001). In opposition to Wang et al.’s findings, Song et al. (2018) found the ff genotype of the *FokI* polymorphism to be associated with a 1.985 times greater risk of myelopathy (*p* = 0.003) [49].

### 3.2. What Are the Genetic Effects on Clinical Severity of DCM?

Seven studies investigated the genetic effects on the clinical severity of DCM, while 11 investigated radiological severity (four studies investigated both). Polymorphisms of 8 genes affected radiological severity, while three genes affected clinical severity. Table 2 presents the full results.

CSM patients with the Val66Met polymorphism of the *BDNF* gene had more severe disease, as assessed by functional survey: worse SF-36 scores for physical functioning and physical health summary than their counterparts without the polymorphism (*p* < 0.05) [53].

Wang et al. (2014) studied the effect of two polymorphisms of the *HIF1A* gene on CSM: 1772C>T and 1790G>A [35]. While the former conferred no significant difference in mJOA score, in the latter patients with the A allele had significantly worse mJOA scores than their G allele counterparts (*p* < 0.001).

Yu et al. (2018) found the TT genotype of the 950T>C polymorphism in the *OPG* gene to be associated with higher mJOA scores and fewer ossified vertebrae (*p* < 0.05); the TT genotype appears to be protective [42].

Wu et al. (2014) investigated four polymorphisms of the *OPN* gene in 187 CSM patients, finding no significant difference of all four polymorphisms on the mJOA score [43].

There was no effect of the Thr420Lys polymorphism of the *VDBP* gene on mJOA score or the number of ossified segments in 318 CSM patients [49]. Similarly, four polymorphisms of the *VDR* gene (*FokI*, *BsmI*, *ApaI*, *TaqI*) were found to have no significant effect on mJOA score in two studies [49,51].

### 3.3. What Are the Genetic Effects on Response to Surgery in DCM?

The polymorphisms of five genes were associated with clinical response to surgery in DCM: *APOE*, *BMP4*, *HIF1A*, *OPN*, and *RUNX2*. The *NPPS* gene was studied for radiological response to surgery. Table 3 presents the results.

In the *APOE* gene, the ε4 allele was associated with an increased risk of poor response to ACDF surgery. In a multivariate model, it was associated with an 8.6 times risk of worsening or no change in mJOA score (*p* = 0.004) [54].

The 6007C>T polymorphism of the *BMP4* gene was associated with greater likelihood of post-surgical improvement of mJOA score (OR 1.53, *p* = 0.002), but the -5826G>A polymorphism had no significant effect (*p* = 0.053) [25].

In the *HIF1A* gene, the 1790G>A polymorphism was also associated with a greater likelihood of post-surgical improvement of the mJOA score (OR 1.55, *p* = 0.024) [35].

In the *OPN* gene, the GG genotype of the −66T>G SNP was found to be associated with worse response to surgical intervention, as assessed by mJOA score (OR 3.62, *p* = 0.007) [43]. Good surgical response was defined as >50% improvement in mJOA score.

Seven polymorphisms of the *RUNX2* gene were investigated for their effect on pre- vs. post-surgical mJOA score. The patients with the CC genotype of the rs16873379 SNP improved less (52.4%) than patients with TT genotype (61.7%), an effect that is mirrored by patients with the AA genotype of the rs1406846 SNP and patients with the CC genotype of the rs2677108 SNP. Patients with the AA genotype of the rs6908650 SNP improved more (66.8%) than their counterparts with the GG genotype (57.4%). The three other polymorphisms had no significant effect on mJOA score improvement [44].

In the NPPS gene, the AA genotype of the A533C polymorphism was associated with a 3.11 times greater likelihood of radiological improvement after surgical intervention. Similarly the IVS20-11delT homozygous T deletion was associated with a 3.35 greater likelihood of improvement. For both polymorphisms, improvement was defined as an increase of <2 mm in the ossified mass of the posterior longitudinal ligament over a mean follow-up length of 3.1 years [41].

## 4. Discussion

The aim of this study was to critically appraise the current evidence on the genetic contribution to DCM, with specific focus on distinguishing spinal column disease from spinal cord disease. Studies were identified evaluating the susceptibility, severity, and responsiveness to surgery in DCM. Studies on spinal column disease focused on the radiological outcomes of OPLL. Evidence was identified for a number of genes, including many in the *TGFβ* superfamily and many known to be associated with bone development.

By further focusing on studies evaluating relationships with clinical function, versus radiological measures, a shortlist of genes that were related to spinal column disease or ‘myelopathy’ and not ‘spondylosis’ was identified: specifically, 12 genes that were associated with susceptibility, three genes with clinical severity, and five genes with response to surgical intervention. Table 4 presents a summary of the evidence for genetic effects on ‘myelopathy’, including GRADE rating for each gene. Across the three focuses of this review (susceptibility, severity, response to surgery), the GRADE rating of quality of evidence is baseline low, as all studies are observational. For all three, the quality of evidence is upgraded due to the large effects across genes, but downgraded due to inconsistency between studies.

### 4.1. Spinal Column Disease: Focus on OPLL

The greatest focus of research to date has been on the bone morphogenetic proteins, a group of multifunctional growth factors that fall within the *TGFβ* superfamily and are involved in cartilage development and the induction of bone formation [55]. Four genes within this family of growth factors have been associated with both altered susceptibilities to bony spinal pathology and altered susceptibility to the development of myelopathy: *BMP2*, *BMP4*, *BMP9*, and *BMPR1A*. The 4A>C SNP in the *BMPR1A* gene is associated with a significantly greater likelihood of radiological OPLL and a significantly greater number of ossified vertebrae [27]. Similarly, the CTCA haplotype of the *BMP9* gene is associated with a significantly increased risk of developing OPLL (OR 2.37), as well as a greater number of ossified vertebrae [26]. In the *BMP4* gene, a haplotype of 7 SNPs is associated with both greater susceptibilities to OPLL and worse disease [24]. Moreover, the 6007C>T SNP in the *BMP4* gene is associated with not only greater likelihood of developing bony pathology and greater severity of radiological disease, but also a greater likelihood of post-operative improvement of the mJOA score [23,25].

The dual role of 6007C>T SNP in the *BMP4* gene merits further discussion. The T allele of the polymorphism was found to be protective for spinal cord disease [25] (AOR 0.51) and it was associated with better outcomes in mJOA score after surgery (AOR 1.53 of being in the ‘improvement’ group). Conversely, Meng et al. found the same T allele to be associated with a greater likelihood of radiological OPLL (OR 1.57) [23]. The contrasting effect of the same allele suggests the effect of the *BMP4* gene is not limited to spinal pathology and the development of bony compression, but it may also influence the spinal cord response to such compression. It is unclear whether this effect is due to an intrinsic effect of *BMP4* on CNS resilience or regeneration, or a treatment artifact that faster compression elicited by the 6007C>T polymorphism giving more severe bony pathology results in faster decompression and better post-operative outcomes. Nonetheless, it is clear that bone morphogenetic protein genes may have extensive influences in the pathogenesis and symptoms of DCM.

Alongside the *BMP* genes, several other genes should be highlighted. In the *NPPS* gene, the C973T polymorphism significantly affected both the susceptibility of OPLL development and the thickness of ossified vertebrae, but notably did not affect the number of ossified vertebrae. 

*NPPS* gene polymorphisms were implicated in post-surgical improvements of spinal column disease affecting the thickness of ossified vertebrae (C973T), while others (IVS15-14T>C) affect the number of ossified vertebrae and others affect both (IVS20-11delT) [41]. 

**Figure 3:**
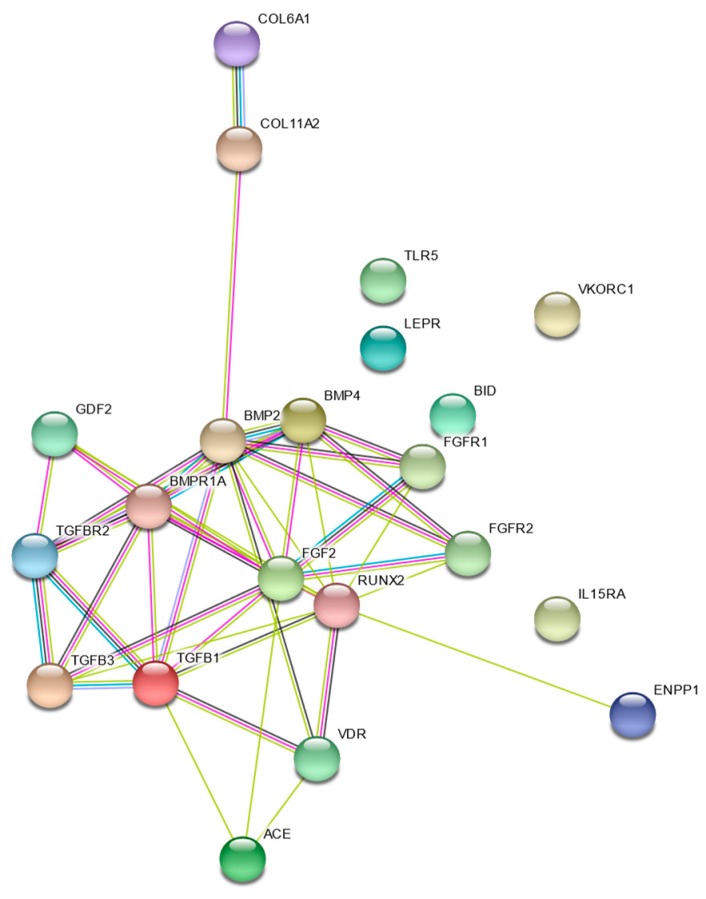
STRING Evidence Network for genes associated with spinal column disease.

Evaluation of the network of genes that were found to be associated with the development of spinal column pathology shows that, while each gene has an independent effect on susceptibility to pathology, there is clear connectedness within and across gene families (Figure 3).

### 4.2. Spinal Cord Disease

The ε4 allele of the *APOE* gene, an allele that is well known for its associations with both cardiovascular disease and Alzheimer’s disease, was associated with both a significantly increased likelihood of DCM development (OR 3.50) [16] and a significantly greater likelihood of failing to gain post-operative improvement (AOR 8.60 no improvement) [54]. However, this effect might not be universal across ethnicities; a study in an Indian population found the ε2 allele to be associated with development of myelopathy (OR 6.69) [17].

The 1790G>A polymorphism of the *HIF1A* gene displayed the opposite effect: it was associated with significantly greater likelihood of DCM development (OR 1.62), and worse disease but a greater likelihood of post-surgical improvement (OR 1.55) [35].

Reductions in Hif1α expression have been shown to be associated with the neuroprotective benefits of hyperbaric oxygen in spinal cord injury mouse models [56]. It is possible that such a mechanism is also the mediator of the *HIF1A* polymorphism’s effect on susceptibility, severity, and post-operative response in DCM.

The *APOE* gene and its product, the apolipoprotein E transporter, are well-known to be involved remyelination, with defective clearance of myelin debris by the transporter limiting the potential for remyelination [57]. In the case of both *HIF1A* and *APOE*, their effects appear to be directly exerted on the cord’s response to bony pathology, rather than via the bony pathology itself. 

There appears to be delineation between genetic factors contributing to the development of bony pathology in the cervical spine, and those contributing to the CNS response to such insult. That an SNP of brain-derived neurotrophic factor (*BDNF*) is associated with the severity of disability (i.e., CNS response to insult) gives further weight to such a distinction [53].

**Figure 4:**
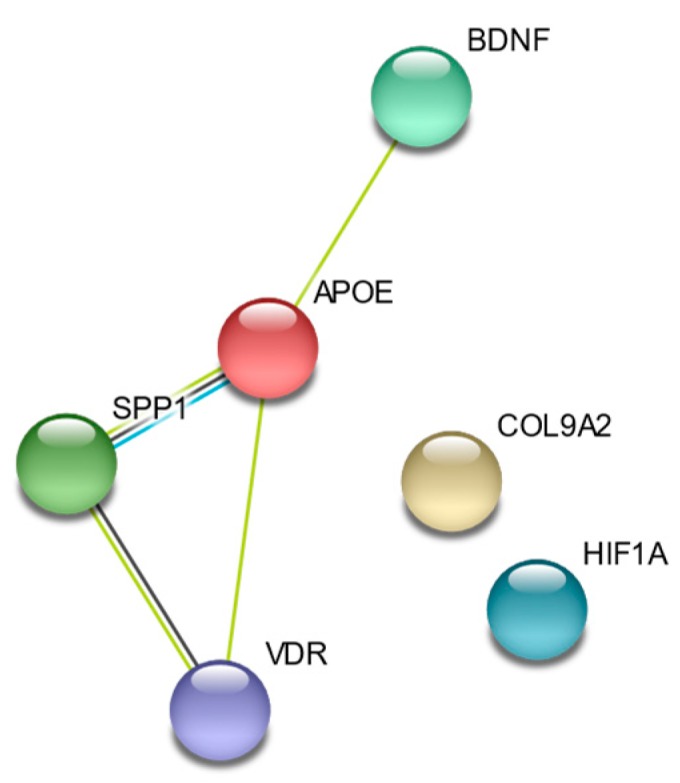
STRING Evidence Network diagram for genes associated with spinal cord disease.

As with genes that are associated with spinal pathology, the genes studied with relation to spinal cord disease have independent, but connected, effects (Figure 4).

### 4.3. Conflicting Evidence

The frequency of conflicting evidence is one striking aspect of much of the work reviewed here. The best example of this is perhaps seen in the *RUNX2* gene; the rs1406846 SNP A allele is associated with 5.67 times greater likelihood of developing DCM in one study [44], but it has no significant effect in a further study using a similar number of participants from the same country [20]. Similarly the 869T>C SNP in the *TGFB1* gene was associated with an odds ratio of 4.50 in one study [45], but a larger, more recent study found no significant effect of the same allele [46], with the result of meta-analysis showing no significant effect. Further examples of conflicting evidence include the IVS20-11delT polymorphism of the *NPPS* gene, in which one study found a significant effect on DCM susceptibility [39], but two others found no significant effect [38,41], while in the IVS15-14T>C polymorphism, two studies found an effect on susceptibility [40,41], with a further study showing no significant effect [33]. Such inconsistency might reflect the relatively small sample sizes of much of the work described here, and it indicates the need for large, well powered genetic investigations. 

### 4.4. Limitations of Current Work

Limitations of the current work on the genetics of DCM are multiple. Firstly, many of the studies that were reviewed in this article scored poorly on the MINORS methodological items assessment [13]. None published information regarding prospective calculation of study size, few reported whether the cases and controls were demographically matched, and some did not report how participants were recruited (e.g., consecutively). As mentioned above, the sample sizes remain in the hundreds rather than thousands, which limits the degree to which their conclusions can be considered valid. Moreover, in reporting the results, many omit odds ratios, instead of reporting only *p*-values, which limits the degree to which such results can be interpreted.

Many of the studies reviewed here focused exclusively on Japanese, Chinese, or South Korean participants, and specifically OPLL. Interestingly, in the *APOE* gene ethnicity appears to result in conflicting genetic effects, with the ε2 allele associated with myelopathy in Indian populations and the ε4 allele associated with myelopathy in Chinese populations [16,17]. It is widely acknowledged that there is a greater prevalence of OPLL within Asian populations, and this might explain their disproportionate representation in the literature [1]. However, without further work across ethnicities, it remains speculation as to whether the conclusions from these studies are globally relevant and across the spectrum of DCM pathologies.

There is significant diversity in the assessment of disease severity between studies. One study used the SF-36 quality of life survey [53], three used the mJOA score [35,43,51] (a clinical score commonly used in DCM research [58,59,60,61]), while others used radiographic measures [19,23,24,26,27,41,44,51]. A similar situation is found within the literature while considering response to surgery, with one study using a cut-off for ‘improvement’ as +1 point on mJOA score [54], some using >50% increase in mJOA score [25,35,43], one using a t-test of % improvement on mJOA between homozygous groups [44], and one paper while using a radiographic definition of disease progression [41]. Such heterogeneity of outcome measures limits the degree to which the effects of genes on severity of DCM and response to surgery can be compared. The removal of surrogate outcome measures and more consistent use of a single form of outcome measure would permit more readily comparable conclusions to be drawn across different studies. We are currently undertaking RECODE DCM, an international consensus process to standardize the reporting of data elements in DCM research, and this would clearly hold benefit here (www.recode-dcm.com) [62]. For the reasons that are outlined above, the GRADE ratings of quality of evidence for each candidate gene were ‘low’ across all genes.

### 4.5. Future Directions

**Figure 5:**
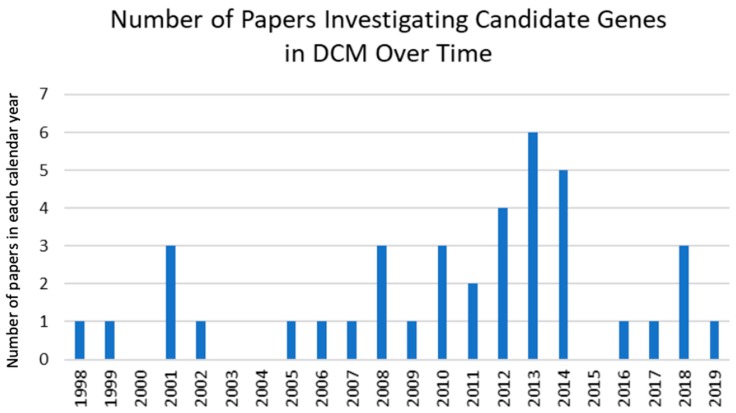
Bar graph of number of papers investigating candidate genes in DCM in each calendar year.

It is clear that interest in this field is building, with increasing numbers of studies focusing on genetic effects in DCM (Figure 5). However, more than half the that are genes reviewed here have been investigated by only a single study, often with small sample sizes, which suggests more intensive work in larger populations is required to further describe the genetic basis of DCM. Furthermore, all of the studies included in this review focused on individual candidate genes. While some considered the effects of haplotypes consisting of several SNPs within a single gene [24,26,29], no work has yet combined SNPs across different genes. Such combinations may exhibit effect sizes of greater magnitude than those in the current body of literature, with potential for such genetic profiles permitting greater personalization of treatment strategies. Future work should also seek to characterize the mechanism by which the genes that were reviewed here exert their effects in the pathobiology of DCM.

## 5. Conclusions

While a number of limitations of the current work do exist, there is clear evidence of genetic effects of single nucleotide polymorphisms and haplotypes in DCM. Some of the genes exert their influence on the development of bony pathology, while others have effects on the spinal cord itself. Further investigation of the genetic basis of DCM requires larger study sizes, using more consistent measures of disease severity and response to surgery. The current evidence base is insufficient for translation to clinical practice for use in prognostication and management, but the potential for genetic profiles to be used in this way may well be realized once greater characterization of the genetic basis of DCM is achieved.

## Figures and Tables

**Table 1:** Susceptibility to radiological or clinical degenerative cervical myelopathy (DCM).

Candidate Gene	Papers Investigating	Study Population Location	No. of Patients	No. of Controls	Matching of Controls	Radiological or Clinical Onset of DCM	Proposed Mechanism	Odds Ratio (Susceptibility)	*p*-Value (Susceptibility)
*ACE*	Kim et al. (2014) [15]	South Korea	95 OPLL	274	Controlled for age and sex in logistic regression models	Radiological	D/D genotype	2.20	0.002
*APOE*	Setzer et al. (2008) [16]	Germany	60 CSM	46	Age, sex. Controls were patients with cervical spondylosis without CSM	Clinical	ε4 allele	3.50	0.008
	Diptiranhan et al. (2019) [17]	India	100 CSM	100		Clinical	ε2 allele vs. ε3 allele	4.4	0.002
							ε2 allele vs. ε4 allele	6.69	0.009
*BID*	Chon et al. (2014) [18]	Korea	157 OPLL	209	Controlled for age and sex in logistic regression models	Radiological	rs8190315 (Ser10 Gly) G allele	2.66	0.005
							rs2072392 (Asp60Asp) C allele	2.66	0.005
*BMP2*	Wang et al. (2008) [19]	China	57 OPLL	135	Age, sex	Radiological	Ser87Ser A/G allele		0.081
							Ser37Ala G allele		<0.001
	Liu et al. (2010) [20]	China	82 (48 OPLL, 12 OLF, 22 both)	118	Age, sex	Radiological	rs1005464 G allele		0.435
	Yan et al. (2013) [21]	China	420 OPLL	506	Age, sex	Radiological	109T>G G allele (Ser37Ala G allele)		<0.001
							570A>T T allele		0.005
	Kim et al. (2014) [22]	South Korea	110 OPLL	211	No. Controls were family members	Radiological	Ser87Ser A/G allele		0.411
							Ser37Ala G allele		0.670
*BMP4*	Meng et al. (2010) [23]	China	179 OPLL	288		Radiological	−5826G>A A allele		0.495
							6007C>T T allele	1.57 (only males)	0.014
	Ren et al. (2012)a [24]	China	450 OPLL	550	Age, sex, BMI, bone mineral density, exercise level, sleeping habit, smoking status, alcohol consumption.	Radiological	rs762642 T>G G allele		0.353
							intron 2 (54422783) G>T T allele		0.868
							rs762643 C>A A allele		0.365
							rs2855530 C>G G allele		0.661
							rs2761884 C>A A allele		0.469
							intron 5 (54419501) G>A A allele		0.684
							intron 5 (54419206) C>T T allele		0.598
							intron 5 (54419150) C>T T allele	3.48	<0.001
							rs10130587 C>G G allele		0.926
							rs35107139 T>G G allele		0.953
							rs2761880 A>G G allele		0.221
							rs74486266 T>C C allele		0.861
							rs17563 C>T T allele	2.22	<0.001
							rs76335800 A>T T allele	1.99	<0.001
							3’-UTR (54416600) A>T T allele		0.190
							rs11335370 T>- deletion		0.608
							intron 6 (54416219) C>T T allele		0.344
							rs59702220 TT>- deletion		0.220
							Haplotype TGGGCTT	2.54	<0.001
	Wang et al. (2013) [25]	China	499 CSM	602	Age, sex, BMI	Clinical	−5826G>A A allele		0.214
							6007C>T T allele	0.51	<0.001
*BMP9*	Ren et al. (2012)b [26]	China	450 OPLL	550	Age, sex, BMI, bone mineral density, exercise level, sleeping habit, smoking status, alcohol consumption.	Radiological	rs3758496		0.301
							rs12252199		0.233
							rs7923671		0.163
							rs75024165	1.82	<0.001
							rs34379100	1.95	0.003
							rs9421799	0.69	0.004
							Haplotype CTCA	2.37	<0.001
*BMPR1A*	Wang et al. (2018) [27]	China	356 OPLL	617	Age, sex	Radiological	−349C>T T allele		<0.001
							4A>C C allele		<0.001
							1327C>T T allele		0.311
							1395G>C		0.586
*COL6A1*	Tanaka et al. (2003) [28]	Japan	342	298	Age	Radiological	rs7671 G>C allele		0.020
							rs2072699 G>A allele		0.958
							intron 2 (+758) C allele		0.019
							rs760437 C>T allele		0.435
							rs754507 A>C allele		0.062
							intron 4 (+20) C allele		0.267
							intron 4 (+37) G allele		0.010
							rs2839076 G>C allele		0.043
							intron 9 (+62) C allele		0.007
							rs2277813 C>G allele		0.057
							rs2277814 G>A allele		0.205
							rs1980982 T>C allele		0.0008
							intron 15 (+39) T allele		0.008
							rs760439 G>A allele		0.048
							rs2850173 C>A allele		0.053
							rs2075893 T>C allele		0.021
							rs2742071 T>C allele		0.219
							rs2850174 T>G allele		0.238
							rs2850175 A>C allele		0.001
							rs2839077 C>T allele		0.005
							rs2276254 A>C allele		0.00009
							rs2276255 A>G allele		0.048
							rs2276256 G>C allele		0.504
							Intron 32 (-29) C allele		0.000003
							rs2236485 G>A allele		0.0002
							rs2236486 A>G allele		0.00005
							rs2236487 A>G allele		0.00006
							rs2236488 C>T allele		0.020
							rs1053312 G>A allele		0.044
							rs1053315 G>A allele		0.040
							exon 35 (+205) T allele		0.677
							rs1053320 C>T allele		0.021
	Kong et al. (2007) [29]	China	183 (90 OPLL, 61 OLF, 32 OPLL and OLF)	155	Sex	Radiological	Promoter (−572) T allele	2.94	0.00003
							intron 32 (-29) C allele	1.89	0.004
	Liu et al. (2010) [20]	China	82 (48 OPLL, 12 OLF, 22 both)	118	Age, sex	Radiological	rs9978314 T allele		0.7618
							rs2276255 G allele		0.7354
	Kim et al. (2014) [22]	South Korea	110 OPLL	211	No. Controls were family members	Radiological	Promoter (−572) T allele		0.282
							intron 33 (+20) G allele		0.625
*COL9A2*	Wang et al. (2012) [30]	China	172 CSM	176	Age, sex, BMI	Clinical	Trp2+ allele	1.78	0.048
							Trp3+ allele		0.087
*COL11A2*	Koga et al. (1998) [31]	Japan	124 paired siblings, 137 OPLL patients	212	No	Clinical	Promoter (−182) C allele		0.0240
							intron 6 (−4) T allele		0.0004
							exon 43 (+24) G allele		0.0210
							exon 46 (+18) T allele		0.0333
	Maeda et al. (2001) [32]	Japan	195 OPLL	187	No	Radiological	intron 6 (−4) T allele	1.99	0.0003
							exon 6 (+28) G allele	1.84	0.0012
	Horikoshi et al. (2006) [33]	Japan	711 OPLL	896	Age	Clinical	rs9277933 (IVS6-4T>A)		0.130
							rs2071025 (IVS29+37C>T)		0.270
*FGF2*	Jun & Kim (2012) [34]	South Korea	157 OPLL	222	Age, sex	Radiological	rs1476217 C allele		0.220
							rs308395 G allele		0.580
							rs3747676 T allele		0.100
*FGFR1*	Jun & Kim (2012) [34]	South Korea	157 OPLL	222	Age, sex	Radiological	rs13317 C allele	2	0.02
*FGFR2*	Jun & Kim (2012) [34]	South Korea	157 OPLL	222	Age, sex	Radiological	rs755793 C allele		0.110
							rs1047100 A allele		0.580
							rs3135831 T allele		0.590
*HIF1A*	Wang et al. (2014) [35]	China	230 CSM	284	Age, sex, BMI	Clinical	1772C>T T allele		0.760
							1790G>A A allele	1.62	<0.001
*IL15RA*	Kim et al. (2011) [36]	South Korea	166 OPLL	230	Age, sex	Radiological	rs2296139 A allele		1.00
							rs2228059 A allele	1.52	0.009
	Guo et al. (2014) [37]	China	235 OPLL	250	Age	Clinical	rs2296139 G allele		0.849
							rs2228059 A allele	1.63	<0.001
*IL18RAP*	Diptiranhan et al. (2019) [17]	India	100 CSM	100		Clinical	rs1420106		>0.05
							rs917997		>0.05
*Leptin receptor*	Tahara et al. (2005) [38]	Japan	156 OPLL	93	Age	Radiological	A861G		0.669
*NPPS*	Nakamura et al. (1999) [39]	Japan	323 OPLL	332	Age	Clinical	IVS20–11delT		0.0029
	Koshizuka et al. (2002) [40]	Japan	180 OPLL	265	Age, sex	Clinical	IVS15-14T>C	3.01	0.022
	Tahara et al. (2005) [38]	Japan	156 OPLL	93	Age	Radiological	IVS20–11delT		0.512
	Horikoshi et al. (2006) [33]	Japan	711 OPLL	896	Age	Clinical	IVS15-14T>C		0.320
	He et al. (2013) [41]	China	95 OPLL	90	Age, sex	Radiological	A533C		0.430
							C973T		<0.001
							IVS15-14T>C		0.026
							IVS20–11delT		0.093
*OPG*	Yu et al. (2018) [42]	China	494 CSM	515		Clinical	950T>C C allele		<0.01
							1181G>C C allele		>0.05
							163A>G G allele		>0.05
*OPN*	Wu et al. (2014) [43]	China	187 CSM	233	Age, sex, BMI	Clinical	−66T>G G allele	1.55	0.002
							−156G/GG GG genotype		0.651
							−443C/T C allele		0.580
*RUNX2*	Liu et al. (2010) [20]	China	82 (48 OPLL, 12 OLF, 22 both)	118	Age, sex	Radiological	rs967588C>T T allele		0.1939
							rs16873379 T>C C allele		0.169
							rs1406846 T>A A allele		0.6646
							rs3749863 A>C C allele		0.8637
							rs6908650 G>A A allele		0.6362
							rs1321075 C>A A allele		0.5255
							rs2677108 T>C C allele		0.6657
							rs16873437 G>T T allele		0.6387
							rs7771889 C>G G allele		0.7854
							rs12333172 C>T T allele		0.8128
							rs9296459 A>G G allele		0.2542
	Chang et al. (2017) [44]	China	80 OPLL	80	Age, sex, BMI, smoking history, alcohol intake	Clinical	rs967588C>T T allele	0.47	0.033
							rs16873379 T>C C allele	0.48	0.033
							rs1406846 T>A A allele	5.67	<0.001
							rs3749863 A>C C allele		0.171
							rs6908650 G>A A allele		0.959
							rs1321075 C>A A allele		0.050
							rs2677108 T>C C allele		0.295
*TGFB1*	Kamiya et al. (2001) [45]	Japan	46 OPLL	273	Age, BMI	Radiological	869T>C CC genotype	4.5	0.0004
	Horikoshi et al. (2006) [33]	Japan	711 OPLL	896	Age	Clinical	IVS2+114G>A A allele		0.330
	Han et al. (2013) [46]	South Korea	98 OPLL	200	Age, sex	Radiological	869T>C CC genotype		0.656
							−509C>T TT genotype		0.931
*TGFB3*	Horikoshi et al. (2006) [33]	Japan	711 OPLL	896	Age	Clinical	IVS1-1284G>C CC genotype	1.46	0.044
*TGFBR2*	Jekarl et al. (2013) [47]	South Korea	21 OPLL	42	None mentioned.	Radiological	445T>A A allele	2.81	0.007
							571G>A A allele	8.73	0.024
							1167C>T T allele		0.888
*TLR5*	Chung et al. (2011) [48]	South Korea	166 OPLL	231	Age, sex	Radiological	rs2072493 G allele		0.457
							rs57441714 C allele		0.457
							rs5744168 T allele		0.543
*VDBP*	Song et al. (2018) [49]	China	318 CSM	282	Age, sex, BMI, smoking	Clinical	Thr420Lys	0.973	0.834
*VDR*	Kobashi et al. (2008) [50]	Japan	63 OPLL	126	Age, sex	Radiological	*FokI* FF genotype	2.33	0.0073
	Wang et al. (2010) [51]	China	154 CSM	156	Age, sex, BMI, desk work time, smoking	Clinical	*FokI* T allele		>0.05
							*BsmI* A allele		>0.05
							*ApaI* A allele	2.88	<0.001
							*TaqI* C allele	4.67	<0.001
	Liu et al. (2010) [20]	China	82 (48 OPLL, 12 OLF, 22 both)	118	Age, sex	Radiological	rs11168287 G allele		0.5933
							rs11574079 A allele	2.68	0.0714
							rs2189480 C allele		0.4197
							rs3847987 C allele		0.6687
							rs12721370 T allele		0.4000
	Song et al. (2018) [49]	China	318 CSM	282	Age, sex, BMI, smoking	Clinical	*FokI* FF genotype	1.461	0.001
*VKORC1*	Chin et al. (2013) [52]	South Korea	98 OPLL	200	Age, sex, hypertension, diabetes mellitus	Radiological	−1639G>A GA genotype	5.22 (female patients only)	0.004 (Non-significant in male/mixed)

**Table 2:** Radiological or clinical severity of DCM.

Candidate Gene	Papers Investigating	Study Population Location	No of Patients	Method of Severity Assessment	Proposed Mechanism	Outcome
*BDNF*	Abode-Iyamah et al. (2016) [53]	USA	10 CSM	Short Form 36 Survey	Val66Met mutation	Met allele subjects had worse scores for physical functioning and social functioning (*p* < 0.05). Met allele subjects had worse ‘physical health summary’ scores (*p* = 0.02).
*BMP2*	Wang et al. (2008) [19]	China	57 OPLL	Number of ossified vertebrae on lateral cervical radiograph (1–7)	Ser87Ser GG genotype	Patients with GG genotype had significantly greater number of ossified vertebrae (*p* < 0.001)
					Ser37Ala GG genotype	No significant difference in number of ossified vertebrae (*p* = 0.113)
*BMP4*	Meng et al. (2010) [23]	China	179 OPLL	Number of ossified vertebrae on lateral cervical radiograph/CT/MRI (1–7)	−5826G>A A allele	No significant difference in number of ossified vertebrae (*p* = 0.324)
					6007C>T T allele	Patients with T allele had significantly greater number of ossified vertebrae (*p* = 0.043)
	Ren et al. (2012)a [24]	China	450 OPLL	Number of ossified vertebrae on lateral cervical radiograph/CT (1–7)	Haplotype TGGGCTT	Patients with the TGGGCTT haplotype had significantly greater number of ossified vertebrae (*p* = 0.002)
*BMP9*	Ren et al. (2012)b [26]	China	450 OPLL	Number of ossified vertebrae on lateral cervical radiograph/CT (1–7)	Haplotype CTCA	Patients with the CTCA haplotype had significantly greater number of ossified vertebrae (*p* = 0.001)
*BMPR1A*	Wang et al. (2018) [27]	China	356 OPLL	Number of ossified vertebrae on lateral cervical radiograph (1–7)	4A>C C allele	Patients with C allele had significantly greater number of ossified vertebrae (*p* < 0.001)
*HIF1A*	Wang et al. (2014) [35]	China	230 CSM	mJOA score	1772C>T T allele	No significant difference in mJOA score (*p* > 0.05)
					1790G>A A allele	Patients with A allele had significantly worse mJOA scores (*p* < 0.001)
*NPPS*	He et al. (2013) [41]	China	95 OPLL	Number of ossified vertebrae on lateral cervical radiograph (1–7)	A533C	No significant difference in number of ossified vertebrae (*p* = 0.363)
					C973T	No significant difference in number of ossified vertebrae (*p* = 0.248)
					IVS15-14T>C	Patients with T allele had significantly greater number of ossified vertebrae (*p* < 0.001)
					IVS20–11delT	Patients homozygous for the T deletion had significantly fewer ossified vertebrae (*p* < 0.001)
				Ossified thickness of cervical vertebrae on lateral radiograph	A533C	No significant difference in ossified thickness of cervical vertebrae (*p* = 0.947)
					C973T	Patients with T allele had significantly thicker ossification of cervical vertebrae (*p* = 0.007)
					IVS15-14T>C	Patients with T alelle had significantly thicker ossification of cervical vertebrae (*p* = 0.017)
					IVS20–11delT	Patients homozygous for the T deletion had significantly less thick ossification of cervical vertebrae (*p* < 0.001)
*OPG*	Yu et al. (2018) [42]	China	494 CSM	mJOA score and number of ossified vertebrae	950T>C	TT genotype associated with higher mJOA scores and fewer ossified cervical vertebrae (*p* < 0.05).
*OPN*	Wu et al. (2014) [43]	China	187 CSM	mJOA score	−66T>G G allele	No significant difference in mJOA score (*p* > 0.05)
					−156G/GG GG genotype	No significant difference in mJOA score (*p* > 0.05)
					−443C/T C allele	No significant difference in mJOA score (*p* > 0.05)
*RUNX2*	Chang et al. (2017) [44]	China	80 OPLL	Number of ossified vertebrae on CT/MRI (1–7)	rs967588C>T T allele	No significant difference in number of ossified vertebrae (*p* = 0.784)
					rs16873379 T>C C allele	Patients with C allele had significantly greater number of ossified vertebrae (*p* = 0.001)
					rs3749863 A>C C allele	No significant difference in number of ossified vertebrae (*p* = 0.129)
					rs6908650 G>A A allele	No significant difference in number of ossified vertebrae (*p* = 0.813)
					rs1321075 C>A A allele	No significant difference in number of ossified vertebrae (*p* = 0.610)
					rs1406846 T>A A allele	Patients with A allele had significantly greater number of ossified vertebrae (*p* = 0.020)
					rs2677108 T>C C allele	Patients with C allele had significantly greater number of ossified vertebrae (*p* = 0.044)
*VDBP*	Song et al. (2018) [49]	China	318 CSM	mJOA score	Thr420Lys	No significant difference in mJOA score (*p* = 0.546)
				Number of ossified vertebrae	Thr420Lys	No significant difference in number of ossified vertebrae (*p* = 0.117)
*VDR*	Wang et al. (2010) [51]	China	154 CSM	Number of segmental lesions on MRI	*FokI* T allele	No significant difference in mean number of segmental lesions (*p* > 0.05)
					*BsmI* A allele	No significant difference in mean number of segmental lesions (*p* > 0.05)
					*ApaI* A allele	No significant difference in mean number of segmental lesions (*p* > 0.05)
					*TaqI* C allele	No significant difference in mean number of segmental lesions (*p* > 0.05)
				mJOA score	*FokI* T allele	No significant difference in mJOA score (*p* > 0.05)
					*BsmI* A allele	No significant difference in mJOA score (*p* > 0.05)
					*ApaI* A allele	No significant difference in mJOA score (*p* > 0.05)
					*TaqI* C allele	No significant difference in mJOA score (*p* > 0.05)
	Song et al. (2018) [49]	China	318 CSM	mJOA score	*FokI* ff genotype	No significant difference in mJOA score (*p* = 0.358)
				Number of ossified vertebrae	*FokI* ff genotype	No significant difference in number of ossified vertebrae (*p* = 0.575)

**Table 3:** Response to surgery in DCM.

Candidate Gene	Papers Investigating	Study Population Location	Number of Patients	Surgery Type	Mean Follow-Up	Method of Assessment of Response to Surgery	Improvement Defined As	Proposed Mechanism	Odds Ratio of No Improvement	Odds Ratio of Improvement	*p*-Value
*APOE*	Setzer et al. (2009) [54]	Germany	60 CSM	ACDF	18.8 months	mJOA score	mJOA score +1	ε4 allele	3.3 (8.6 in multivariate model)	-	**0.002** (0.004 multivariate model)
*BMP4*	Wang et al. (2013) [25]	China	499 CSM	Anterior cervical corpectomy and fusion	12 months	mJOA score	>50% improvement in mJOA score	−5826G>A A allele	-	-	0.053
								6007C>T T allele	-	1.53	**0.002**
*HIF1A*	Wang et al. (2014) [35]	China	230 CSM	Anterior cervical corpectomy and fusion	24 months	mJOA score	>50% improvement in mJOA score	1790G>A A allele	-	1.55	**0.024**
*NPPS*	He et al. (2013) [41]	China	95 OPLL		3.1 years	Progression of OPLL ossification on lateral radiograph	<2 mm increase in ossified mass of PLL	A533C AA genotype	-	3.11	**0.029**
								C973T	-	-	0.935
								IVS15-14T>C	-	-	0.836
								IVS20–11delT homozygous T deletion	-	3.35	**0.007**
*OPN*	Wu et al. (2014) [43]	China	187 CSM	Anterior cervical corpectomy and fusion	24 months	mJOA score	>50% improvement in mJOA score	−66T>G GG genotype	3.62	-	**0.007**
*RUNX2*	Chang et al. (2017) [44]	China	80 OPLL	Laminoplasty	12 months	mJOA score	% improvement in mJOA score	rs967588C>T T allele	-	-	>0.05
								rs16873379 T>C C allele	-	-	**<0.05**
								rs3749863 A>C C allele	-	-	>0.05
								rs6908650 G>A A allele	-	-	**<0.05**
								rs1321075 C>A A allele	-	-	>0.05
								rs1406846 T>A A allele	-	-	**<0.05**
								rs2677108 T>C C allele	-	-	**<0.05**

**Table 4:** Summary of candidate genes affecting myelopathy (i.e., ***clinical*** onset/severity/response to surgery rather than radiological). Colour coded for evidence level (green: unconflicted evidence, amber: conflicting evidence, red: no evidence or not yet investigated). GRADE rating of quality of evidence given for each candidate gene—baseline quality low (all observational studies); gene-specific upgrade/downgrade comments in parentheses.

Candidate Gene	Papers Investigating	Susceptibility to Myelopathy	Severity of Myelopathy	Post-Operative Response	GRADE Rating
*APOE*	Setzer et al. (2008) [16] Setzer et al. (2009) [54]	ε4 allele: OR 3.50, *p* = 0.008		ε4 allele: OR of no improvement 3.3 (8.6 in multivariate model), *p* = 0.002 (*p* = 0.004)	Low (small sample size, inconsistency across ethnicities)
	Diptiranhan et al. (2019)	ε2 allele: OR 6.69, *p* = 0.009			
*BDNF*	Abode-Iyamah et al. (2016) [53]		Val66Met: Met allele subjects had worse scores for physical functioning (*p* < 0.05), social functioning (*p* < 0.05 and ‘physical health summary’ (*p* = 0.02) on SF-36 survey.		Low (single study, very small sample size)
*BMP4*	Wang et al. (2013) [25]	6007C>T T allele: OR 0.51, *p* < 0.001		6007C>T T allele: OR of improvement 1.53, *p* = 0.002	Low (inconsistency across studies, inconsistency between CSM and OPLL studies)
*COL9A2*	Wang et al. (2012) [30]	Trp2+ allele: OR 1.78, *p* = 0.048			Low (single study, small sample size)
*COL11A2*	Koga et al. (1998) [31]	Promoter (−182) C allele (*p* = 0.0240); Intron 6 (−4) T allele (*p* = 0.0004); Exon 43 (+24) G allele (*p* = 0.0210); Exon 46 (+18) T allele (*p* = 0.0333)			Low
*HIF1A*	Wang et al. (2014) [35]	1790G>A A allele: OR 1.62, *p* < 0.001	1790G>A A allele associated with worse mJOA scores (*p* < 0.001)	1790G>A A allele: OR of improvement 1.55, *p* = 0.024	Low (single study)
*IL15RA*	Guo et al. (2014) [37]	rs2228059 A allele: OR 1.63, *p* < 0.001			Low
*NPPS*	Nakamura et al. (1999) [39]	IVS20-11delT: *p* = 0.0029			Low (inconsistency across studies)
	Koshizuka et al. (2002) [40]	IVS15-14T>C: OR 3.01, *p* = 0.022 NB. Horikoshi et al. (2006) find *p* = 0.320.			
*OPG*	Yu et al. (2018) [42]	950T>C C allele: *p* < 0.01	950T>C TT genotype associated with higher mJOA scores and fewer ossified vertebrae (*p* < 0.05)		Low (single study)
*OPN*	Wu et al. (2014) [43]	−66T>G G allele: OR 1.55, *p* = 0.002	No significant difference in mJOA score (*p* > 0.05).	-66T>G GG genotype: OR of no improvement 3.62, *p* = 0.007	Low (single study)
*RUNX2*	Chang et al. (2017) [44]	rs967588C>T T allele: OR 0.47, *p* = 0.033; rs16873379T>C C allele: OR 0.48, *p* = 0.033; rs1406846T>A A allele: OR 5.67, *p* < 0.001		rs16873379T>C C allele: *p* < 0.05; rs6908650G>A A allele: *p* < 0.05; rs1406846T>A A allele: *p* < 0.05; rs2677108T>C C allele: *p* < 0.05	Low
*TGFB3*	Horikoshi et al. (2006) [33]	IVS1-1284G>C CC genotype: OR 1.46, *p* = 0.044			Low (single study)
*VDR*	Wang et al. (2010) [51]	*ApaI* A allele: OR 2.88, *p* < 0.001; *TaqI* C allele: OR 4.67, *p* < 0.001	No significant difference in mJOA score (*p* > 0.05).		Low (inconsistency across studies)
	Song et al. (2018) [49]	*FokI* ff genotype: OR 1.985, *p* = 0.003	No significant difference in mJOA score or number of ossified vertebrae (*p* > 0.05)		

## Supplementary Materials

The following are available online at https://www.mdpi.com/2077-0383/9/1/282/s1, Data S1: search strategy used for MEDLINE and EMBASE databases. Data S2: search strategy used for Cochrane and HuGENet databases. Data S3: PRSIMA checklist.
